# Meiotic activation of Mitf/TFEB declines with age in the *Drosophila* testis

**DOI:** 10.17912/micropub.biology.001509

**Published:** 2025-02-07

**Authors:** Tyler J. Butsch, Alyssa E. Johnson, K. Adam Bohnert

**Affiliations:** 1 Biological Sciences, Louisiana State University, Baton Rouge, Louisiana, United States

## Abstract

Lysosome activity regulates germline development in multiple species. In the *Drosophila* testis, lysosomes activate as germ cells exit mitosis and enter meiosis. Notably, reduced activity of germ-cell lysosomes, which is seen during aging, leads to fewer viable sperm. Here, we investigated the activity of Mitf/TFEB, a master regulator of lysosome biogenesis, during *Drosophila* spermatogenesis. We discovered that Mitf activity was upregulated in meiotic-stage spermatocytes, consistent with the lysosome-activation pattern. However, Mitf activity in spermatocytes declined in older males, concurrent with reduced expression of a Mitf-targeted V-ATPase component. These findings provide insight into the regulation of upstream lysosome controls during spermatogenesis.

**Figure 1. Developmental and age-related regulation of Mitf activity in the Drosophila testis:**
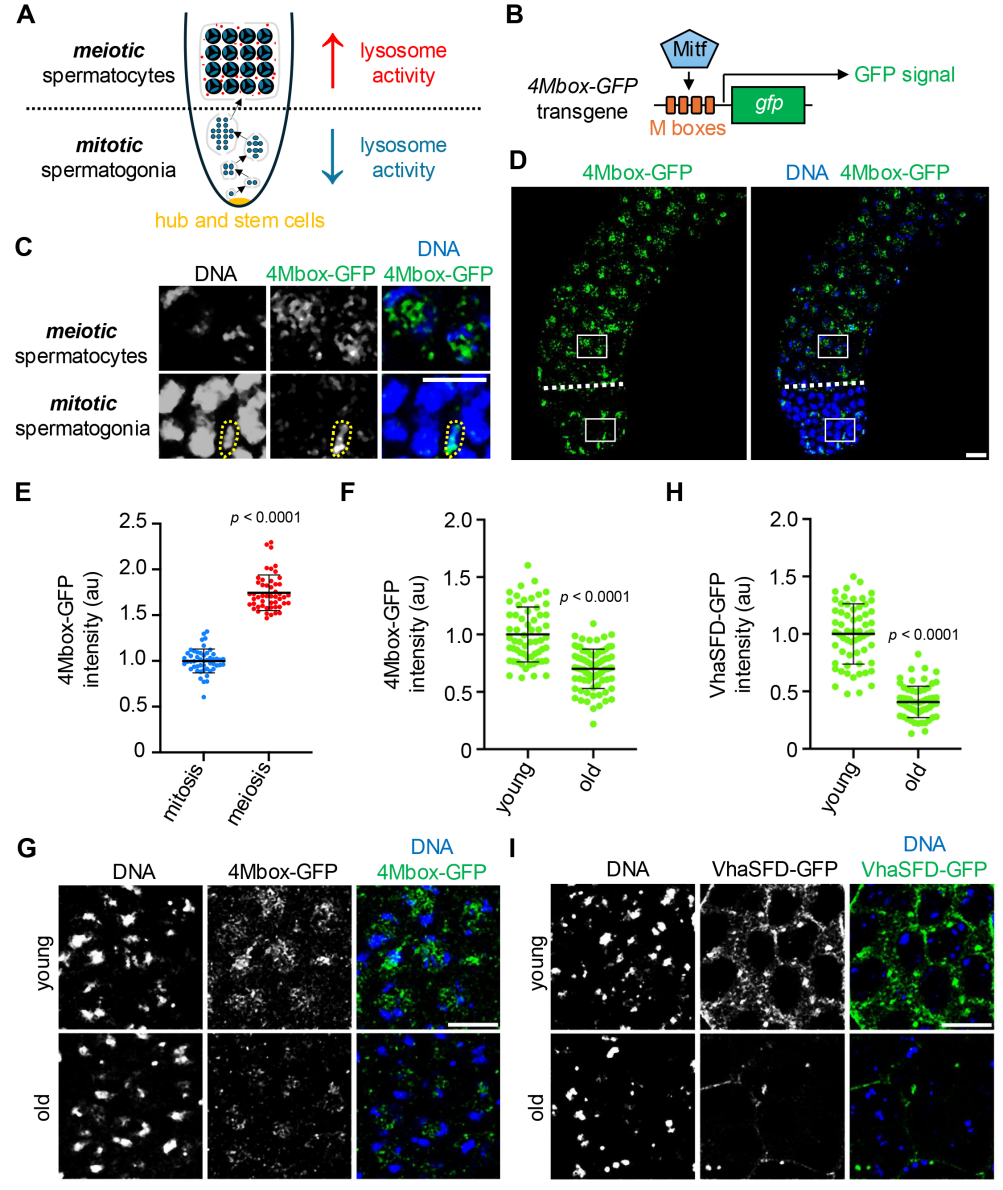
**A** . Schematic of the *Drosophila* testis. Stem cells reside in the hub (yellow) at the apical tip. Germline stem cells produce a spermatogonium, which undergoes four mitotic divisions. Subsequently, germ cells enter meiosis, producing 16 spermatocytes. Somatic cyst cells (gray lines) encapsulate developing germ cells within individual cysts. Lysosomes (red) activate at meiosis. **B** . Schematic of the *4Mbox-GFP* transgene. When present and active, Mitf binds to repetitive Mboxes, inducing GFP expression. **C, D.** Representative images of DNA (Hoechst) and 4Mbox-GFP (Mitf activity reporter) in the young-adult *Drosophila* testis. Enlarged regions given for spermatogonia and spermatocytes in C are outlined in D. A cyst cell nucleus is outlined in C. The mitosis-to-meiosis transition is demarcated by a dashed line in D. **E.** Quantification of 4MBox-GFP intensities in mitotic spermatogonia ( *n* =50 spermatogonia from 10 total testes) and meiotic spermatocytes ( *n* =50 spermatocytes from 10 total testes) in young adult males. Mean ± S.D. *p* < 0.0001, Mann-Whitney U-test. **F.** Quantification of 4Mbox-GFP intensities in spermatocytes of young ( *n* =60 spermatocytes from 12 total testes) and old adult males ( *n* =80 spermatocytes from 16 total testes). Mean ± S.D. *p* < 0.0001, Welch's t-test. **G.** Representative images of DNA (Hoechst) and 4Mbox-GFP (Mitf activity reporter) in spermatocytes of young and old adult males. **H.** Quantification of VhaSFD-GFP intensities in spermatocytes of young ( *n* =60 spermatocytes from 12 total testes) and old adult males ( *n* =65 spermatocytes from 13 total testes). Mean ± S.D. *p* < 0.0001, Welch's t-test. **I.** Representative images of DNA (Hoechst) and VhaSFD-GFP in spermatocytes of young and old adult males. Bars, 20 µm.

## Description

Lysosomes are acidic, membrane-bound organelles that serve various metabolic, signaling, and degradative functions in eukaryotic cells (Ballabio and Bonifacino, 2020) . Emerging evidence indicates that lysosome activation may help to define, and ultimately drive, developmental transitions (Butsch et al., 2022; Leeman et al., 2018; Villegas et al., 2019; Xie et al., 2019); linking lysosome activation to specific developmental time-points may provide a switch-like mechanism to rewire cellular homeostasis at key stages, thereby promoting developmental maturation. As an example, we recently reported that lysosomes activate at the mitosis-to-meiosis transition during *Drosophila* spermatogenesis ( Figure 1A ) (Butsch et al., 2022) . At the meiotic spermatocyte stage, lysosome activity is important to stabilize expanding cell membranes, and loss of lysosome function, brought about by experimental means or naturally as a result of aging, leads to pronounced phenotypic defects, including germ-cell multinucleation (Butsch et al., 2022) . Clarifying how lysosome regulators interface with the spermatogenesis program may indicate elements of control and hint at potential changes causing age-related reproductive dysfunction.

Transcription factor EB (TFEB) is a master regulator of lysosome gene expression and lysosome function in multiple systems and contexts (Sardiello et al., 2009; Settembre et al., 2011) . The *Drosophila* TFEB homolog Mitf controls the expression of all 15 vacuolar ATPase (V-ATPase) components (Zhang et al., 2015) , which support lysosome acidification and activation (Forgac, 2007; Ohkuma et al., 1982) . In the *Drosophila* germline, gene expression has been reported to generally increase as spermatocytes enter meiosis (Fuller, 1998; Lin et al., 1996; White-Cooper et al., 1998) . Our own observations have confirmed that VhaSFD, a V-ATPase protein, is more strongly expressed in meiotic spermatocytes than in mitotic spermatogonia (Butsch et al., 2022) . These considerations inspired us to further investigate whether Mitf/TFEB activity is subject to regulation in the *Drosophila* testis, potentially in ways reflecting the pattern of lysosome activation we previously described.

To visualize Mitf activity in the *Drosophila* testis, we used a Mitf activity reporter, 4Mbox-GFP (Zhang et al., 2015) . This reporter takes advantage of defined Mitf binding sites (Mboxes), which are cloned immediately upstream of a *gfp* transgene ( Figure 1B ). 4Mbox-GFP provides a readout of Mitf activity based on GFP fluorescence; the level of GFP expression correlates with Mitf binding and, hence, Mitf activity (Zhang et al., 2015) . Near the apical tip of the testis, GFP was detected in somatic cyst cells ( Figure 1C,D ), which surround germ cells and support their development (Zoller and Schulz, 2012) . However, mitotic spermatogonia were devoid of GFP signal ( Figure 1C,D ). This suggests that there is little Mitf activity in mitotic germ cells, which we previously showed lack activated lysosomes (Butsch et al., 2022) . More distally, in meiotic spermatocytes, we detected robust GFP signal, which was concentrated in regions separate from condensed chromatin ( Figure 1C,D ). Mean fluorescence intensity was heightened in meiotic spermatocytes relative to mitotic spermatogonia ( Figure 1E ). Thus, we conclude that Mitf activity is upregulated as germ cells enter meiosis, consistent with our previous report of lysosome activation at this developmental time-point.

Because we had previously also found that lysosome activity declines with age in *Drosophila* spermatocytes (Butsch et al., 2022) , we hypothesized that Mitf activity may likewise decline in meiotic-stage germ cells as *Drosophila* males aged. Indeed, this was the case; 4Mbox-GFP signal was visibly dimmer and measurably lower in meiotic spermatocytes of 30-day-old adult males compared to meiotic spermatocytes of 1-day-old adult males ( Figure 1F,G ). Additionally, we found that expression of a Mitf-regulated V-ATPase component, VhaSFD (Zhang et al., 2015) , was also significantly lower in spermatocytes of older testes compared to younger testes ( Figure 1H,I ). These data indicate that the Mitf signaling axis, which acts upstream of lysosome activation, deteriorates with age in the *Drosophila* testis and may contribute to the age-related decline in germline lysosome activity.

Collectively, our findings demonstrate that Mitf activity shows developmental patterns similar to lysosome activation during *Drosophila* spermatogenesis, and Mitf activity also naturally decreases with age. It is currently unclear whether Mitf activity is required to support the mitosis-to-meiosis transition or if Mitf activation occurs after cells commit to entering the spermatocyte stage. We and others have previously found that some V-ATPase components are required for the mitosis-to-meiosis transition (Butsch et al., 2022; Varga et al., 2022) , and thus it is conceivable that Mitf activation is likewise a prerequisite. How Mitf activity in the germline is molecularly repressed during mitosis but triggered at meiosis remains an open question. The tumor suppressor PDCD4 has been shown to repress TFEB translation in mice (Chen et al., 2021) , and, interestingly, the *Drosophila* PDCD4 homolog is expressed in *Drosophila* spermatogonia but not in spermatocytes (Cash and Andrews, 2012) , hinting at one potential mechanism based on Mitf protein expression. It will be informative to clarify whether Mitf protein expression mirrors Mitf activity patterns in the testis, or if changes to Mitf co-factors, rather than protein levels, better explain the observed developmental patterns in Mitf activity. Why Mitf activity is more constitutive in somatic cysts cells is also unknown but may help to sustain their support functions throughout development. Remarkably, we have observed lysosome activation at meiotic entry in developing oocytes (Bohnert and Kenyon, 2017; Samaddar et al., 2021) , suggesting that this event may be developmentally conserved in the germline across sexes. It will be important to determine how lysosome regulators such as Mitf/TFEB are regulated at meiotic entry during oogenesis to clarify if similar principles apply. An additional significant task for the future is to identify molecular causes of the age-related reduction in Mitf activity in the testis. Potentially, this may involve mTOR signaling, which can inhibit the transcriptional activity of TFEB homologs (Martina et al., 2012) and is upregulated in the *Drosophila* testis with age (Butsch et al., 2022) . Such future analyses may reveal entry points to counteract age-related reproductive dysfunction and enhance gamete health in older males.

## Methods

**Fly husbandry and aging**

Flies were maintained at 25°C in a 12:12 light:dark cycle on standard cornmeal/agar food [6% (w/v) cornmeal (VWR, 75860-346), 1.5% (w/v) yeast (Genesee Scientific, 62-107), 1% (w/v) agar (Genesee Scientific, 66-105), 8% (v/v) molasses (VWR, 75860-374), 0.8% (v/v) Tegosept (Fisher Scientific, NC0238407), 0.24% (v/v) propionic acid (Fisher Scientific, BPA258500), and 0.02% (v/v) phosphoric acid (Sigma, PX09956)]. 4MBox-GFP (Francesca Pignoni, Upstate Medical University) and VhaSFD-GFP (BDSC #6840) strains were previously generated and partly described (Butsch et al., 2022; Kelso et al., 2004; Morin et al., 2001; Zhang et al., 2015) . For aging experiments, flies were collected less than 24 hours after eclosing and aged in vials on standard cornmeal/agar food at 25°C. At most, 30 flies were placed into a single vial to avoid overcrowding; vials included both males and females, such that males would continue to mate while aging. Flies were transferred to fresh food every 7-8 days. Once flies reached the appropriate age, testes were dissected and imaged. “Young” animals were analyzed at day 1 of adulthood, and “old” animals were analyzed at day 30 of adulthood.

**Immunostaining and microscopy**

Testes were dissected in 1X phosphate-buffered saline (PBS) [137 mM NaCl (Fisher Scientific, S271-10), 2.7 mM KCl (Fisher Scientific, P217-500), 10 mM Na _2_ HPO _4_ (Fisher Scientific, S374-1), 1.8 mM KH _2_ PO _4_ (Fisher Scientific, P285-500)] and then immediately fixed in 4% paraformaldehyde diluted in PBS (VWR, AAJ61899-AK). Testes were washed three times in PBT [1X PBS, 0.1% Tween-20 (VWR, 0777-1L)], then incubated in blocking buffer [3% Bovine Serum Albumin (Fisher Scientific, BP9703-100) in 1X PBS] for at least 1 hour at room temperature. Testes were incubated with the primary antibody (rabbit anti-GFP; Invitrogen, A-21311) diluted 1:1000 in blocking buffer containing 2% Triton X-100 (Fisher Scientific, BP-151). The next day, testes were washed five times with PBT prior to applying the secondary antibody (goat anti-rabbit 488; Invitrogen, A-11034) at a 1:500 dilution. Testes were incubated with the secondary antibody for at least 3 hours at room temperature in the dark. After the secondary antibody solution was removed, testes were washed five times with PBT. 1 µM Hoechst 33342 (Invitrogen, H21492) was incubated in the first wash to stain DNA. Testes were mounted in Vectashield antifade mounting medium (Fisher Scientific, NC1864755) prior to imaging.

Images were acquired using an inverted Leica SP8 confocal microscope, equipped with a 40x objective (NA 1.30) and a white-light laser. Images were processed using Leica LAS X software, and quantifications were performed using Fiji (NIH) on 8-bit images.

**Quantification of fluorescence intensities at different germ-cell stages**

We used Fiji (NIH) to quantify fluorescence intensities of each marker (4Mbox-GFP or VhaSFD-GFP) at the spermatogonia or spermatocyte stage. Briefly, we outlined the region (i.e., germ cell) of interest using the Freehand selection tool, then measured the mean fluorescence intensity using the Analyze>Measure function. Germ-cell stage was determined by chromatin morphology as previously described (Cenci et al., 1994) . In spermatogonia, Hoechst signal is bright and fills the entire nucleus. In spermatocytes, Hoechst signal is dimmer, and bivalents occupy distinct locations in the nucleus, forming a tri-lobed structure.

**Statistical analyses**

Information on sample size and statistics is provided in figure legends where applicable. Data normality was tested via the D'Agostino-Pearson normality test in combination with Q-Q plots prior to performing follow-up statistical analyses using GraphPad Prism software. Welch's unpaired t-test was used when unpaired data for two groups were normally distributed, but standard deviation was not equal. The Mann-Whitney U-test was used when unpaired data for two groups were not normally distributed.

## Reagents

**Table:** 

**Strain**	**Species**	**Source**
4Mbox-GFP	*Drosophila melanogaster*	Francesca Pignoni, Upstate Medical University
VhaSFD-GFP	*Drosophila melanogaster*	Bloomington *Drosophila* Stock Center #6840

**Table:** 

**Antibody**	**Animal**	**Company & Catalog Number**
anti-GFP, Alexa Fluor ^TM^ 488	rabbit	Invitrogen, A-21311
anti-rabbit IgG, Alexa Fluor ^TM^ 488	goat	Invitrogen, A-11034
